# Rolling circle amplification-driven encoding of different fluorescent molecules for simultaneous detection of multiple DNA repair enzymes at the single-molecule level†
†Electronic supplementary information (ESI) available. See DOI: 10.1039/d0sc01652g


**DOI:** 10.1039/d0sc01652g

**Published:** 2020-05-18

**Authors:** Chen-chen Li, Hui-yan Chen, Juan Hu, Chun-yang Zhang

**Affiliations:** a College of Chemistry , Chemical Engineering and Materials Science , Collaborative Innovation Center of Functionalized Probes for Chemical Imaging in Universities of Shandong , Key Laboratory of Molecular and Nano Probes , Ministry of Education , Shandong Provincial Key Laboratory of Clean Production of Fine Chemicals , Shandong Normal University , Jinan 250014 , China . Email: cyzhang@sdnu.edu.cn ; Email: juanhu@sdnu.edu.cn ; Fax: +86 0531-82615258 ; Tel: +86 0531-86186033

## Abstract

DNA repair enzymes (*e.g.*, DNA glycosylases) play a critical role in the repair of DNA lesions, and their aberrant levels are associated with various diseases. Herein, we develop a sensitive method for simultaneous detection of multiple DNA repair enzymes based on the integration of single-molecule detection with rolling circle amplification (RCA)-driven encoding of different fluorescent molecules. We use human alkyladenine DNA glycosylase (hAAG) and uracil DNA glycosylase (UDG) as the target analytes. We design a bifunctional double-stranded DNA (dsDNA) substrate with a hypoxanthine base (I) in one strand for hAAG recognition and an uracil (U) base in the other strand for UDG recognition, whose cleavage by APE1 generates two corresponding primers. The resultant two primers can hybridize with their respective circular templates to initiate RCA, resulting in the incorporation of multiple Cy3-dCTP and Cy5-dGTP nucleotides into the amplified products. After magnetic separation and exonuclease cleavage, the Cy3 and Cy5 fluorescent molecules in the amplified products are released into the solution and subsequently quantified by total internal reflection fluorescence (TIRF)-based single-molecule detection, with Cy3 indicating the presence of hAAG and Cy5 indicating the presence of UDG. This strategy greatly increases the number of fluorescent molecules per concatemer through the introduction of RCA-driven encoding of different fluorescent molecules, without the requirement of any specially labeled detection probes for simultaneous detection. Due to the high amplification efficiency of RCA and the high signal-to-ratio of single-molecule detection, this method can achieve a detection limit of 6.10 × 10^–9^ U mL^–1^ for hAAG and 1.54 × 10^–9^ U mL^–1^ for UDG. It can be further applied for simultaneous detection of multiple DNA glycosylases in cancer cells at the single-cell level and the screening of DNA glycosylase inhibitors, holding great potential in early clinical diagnosis and drug discovery.

## Abstract 2

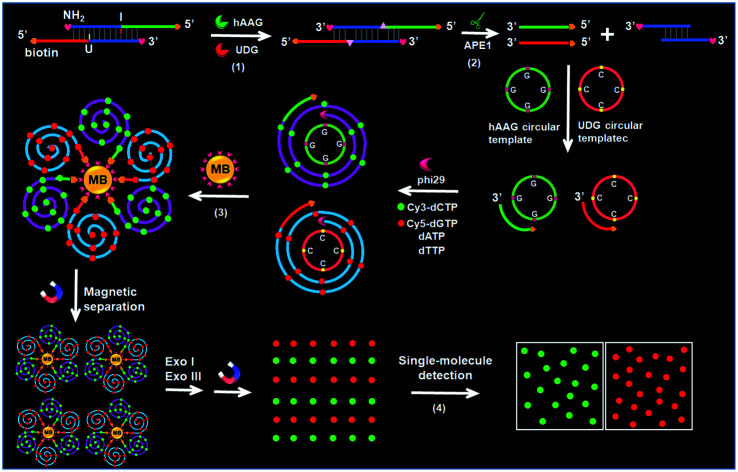
Integration of single-molecule detection with rolling circle amplification-driven encoding of different fluorescent molecules enables simultaneous detection of multiple DNA repair enzymes.

## Introduction

The human genome sequence provides the underlying code for human biology, and the maintenance of genomic integrity is essential for all eukaryotes.^1^,^2^ DNA damage is a natural hazard of life, and the most common DNA lesions are base, sugar, and single-strand break damages resulting from oxidation, alkylation, deamination, and spontaneous hydrolysis.^3^–^5^ To counteract the deleterious effect of DNA lesions, cells have involved multiple repair mechanisms such as base-excision repair (BER), nucleotide excision repair, mismatch repair and double-strand DNA break repair. BER pathway is initiated by DNA glycosylases which recognize the damaged/mismatched bases and excise them from DNA by hydrolyzing the *N*-glycosidic bond between the base and the sugar phosphate backbone of DNA to generate an abasic site, whereas the lyase activity nicks the DNA.^6^–^8^ All mammals express multiple DNA glycosylases to maintain genome stability.^2^ These glycosylases not only have broad substrate specificity, but also have unique specificity.^9^ DNA glycosylases have been associated with both individual and population disease susceptibility,^8^,^10^,^11^ and the aberrant level of DNA glycosylase in human cells may cause the malfunction of base excision repair and eventually various diseases.^11^–^17^ Both human alkyladenine DNA glycosylase (hAAG) and uracil DNA glycosylase (UDG) are glycosylases produced by human body, and their abnormal expressions are closely related to a variety of diseases. For example, hAAG activity in peripheral blood mononuclear cells from lung cancer patients is higher than that in healthy persons,^15^ suggesting that it may become a potential biomarker for lung cancer diagnosis. UDG plays pivotal roles in maintaining genomic integrity, and it is closely associated with the etiology of various diseases including cancer, aging and neurodegenerative diseases.^11^,^13^,^14^ Especially, some diseases exhibit multiple DNA glycosylation enzyme abnormalities.^15^ Therefore, the simultaneous detection of multiple DNA glycosylases will benefit the study of DNA damage repairing process and early clinical diagnosis. The gel-electrophoresis coupled with radioactive labeling is regarded as the gold standard method for DNA glycosylase (*e.g.*, UDG^18^ and hAAG^19^,^20^) analysis, but it suffers from time-consuming procedures, poor sensitivity, and the hazardous radiation.^18^–^20^ Alternatively, some new strategies including electrochemical method,^21^ fluorescent method,^22^–^26^ and imaging method^27^ have been developed for DNA glycosylase assay, but they involve the time-consuming and labour-tedious steps (*e.g.*, graphene-modified electrode fabrication), expensive and complicated probe design (*e.g.*, external labeling with a fluorophore and a quencher), high background signals due to the incomplete quenching (*e.g.*, the fluorescent nucleotide analogs 2-aminopurine and pyrrolo-dC as the fluorophores with DNA molecules as the intrinsic quenchers), and low sensitivity.^21^–^26^ In addition, the reported RCA-based methods enable the detection of only a single type of DNA glycosylases.^28^–^34^ Therefore, it is highly desirable to develop a sensitive method for simultaneous detection of multiple DNA glycosylases.

The polymerase chain reaction (PCR) allows for the identification of specific nucleic acids far below the detection limit of other techniques, but it requires complex thermocycling to mediate denaturation, annealing, and subsequent extension.^35^ Isothermal amplification of nucleic acids (*e.g.*, rolling circle amplification (RCA)) as an alternative amplification technique enables rapid and effective amplification at constant temperature.^35^–^44^ RCA may generate very long single-stranded DNAs (ssDNAs) with tandem repeats, and it can achieve approximately 10^3^-fold amplification within 1 h.^35^,^45^–^47^ The visualization and analysis of RCA products usually use either SYBR green I as the fluorescence label^45^ or the fluorescence-labeled oligonucleotide probe.^46^ SYBR Green I is a DNA intercalating dye that binds dsDNA,^45^ but it has several limitations including the concentration-dependent inhibition of PCR, preferential binding to the GC-rich sequences, the promotion of nonspecific amplification, and the detection of only a single type of target due to the use of a single fluorophore, false positive signals due to its binding to any dsDNAs including nonspecific dsDNA sequences.^48^ Theoretically, the fluorescently labeled oligonucleotide probes allow for single fluorescent molecule per concatemer through the hybridization of oligonucleotide probe with RCA product, and their applications for multiplexed assay needs extra templates and specially labeled detection probes.^46^ Thus, the development of a simultaneous sensitive detection method still remains a great challenge.

To improve both the detection specificity and the multiplexed capability, we develop a sensitive method for simultaneous detection of multiple DNA glycosylases based on the integration of single-molecule detection with RCA-driven encoding of different fluorescent molecules. In comparison with the conventional ensemble fluorescence measurements, the single-molecule detection has remarkable advantages of high sensitivity, low sample consumption, and high signal-to-noise ratio.^49^–^55^ Owing to the high amplification efficiency of RCA and the high signal-to-ratio of single-molecule detection, our method enables simultaneously sensitive detection of multiple DNA glycosylases with a detection limit of 6.10 × 10^–9^ U mL^–1^ for hAAG and 1.54 × 10^–9^ U mL^–1^ for UDG. One significant advantage of our method is that it greatly increases the number of fluorescent molecules per concatemer through the introduction of RCA-driven encoding of different fluorescent molecules, without the requirement of specially labeled detection probes for simultaneous detection, greatly simplifying the experimental procedures and improving the sensitivity. Moreover, our method can be applied for the simultaneous detection of multiple DNA glycosylases in cancer cells at the single-cell level and the discrimination of normal cells from cancer cells. It can be further applied for the analysis of enzyme kinetic parameters and the screening of DNA glycosylase inhibitors, holding great potential in biomedical research, clinical diagnosis and drug discovery.

## Results and discussion

### Principle of simultaneous detection of hAAG and UDG

**Scheme 1:**
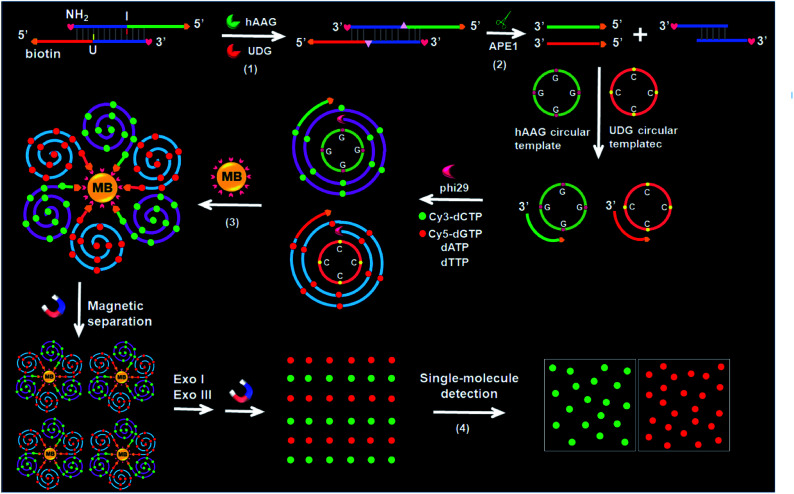
Schematic illustration of the simultaneous detection of multiple DNA repair enzymes by the integration of RCA with single-molecule detection. This strategy involves four steps: (1) specific excision of dsDNA substrate by hAAG and UDG, (2) the hybridization of primers with circular templates and the subsequent RCA reaction, (3) magnetic separation and the cleavage of amplified products by Exonucleases I and III to release fluorescent molecules, and (4) single-molecule detection of the released fluorescent molecules.

The principle of the integration of RCA with single-molecule detection for the simultaneous detection of multiple DNA glycosylases is illustrated in Scheme 1. This assay involves a bifunctional double-stranded DNA (dsDNA) substrate and two circular templates for hAAG and UDG, respectively. We designed an hAAG probe (green + blue color, Scheme 1) modified with one hypoxanthine base (I) at the 22nd base from the 5′ end and a UDG probe (red + blue color, Scheme 1) modified with one uracil base (U) at the 22nd base from the 5′ end, with 5′ and 3′ ends of both two probes being modified with biotin and NH_2_ for preventing the nonspecific amplification, respectively. The hybridization of two probes forms a bifunctional dsDNA substrate for hAAG and UDG. This assay involves four steps: (1) specific excision of dsDNA substrate by hAAG and UDG, (2) the hybridization of primers with circular templates and the subsequent RCA reaction, (3) magnetic separation and the cleavage of amplified products by Exonucleases I and III to release fluorescent molecules, and (4) single-molecule detection of fluorescent molecules by total internal reflection fluorescence (TIRF) microscopy. When hAAG is present, it can specifically recognize I:T base pairs in the dsDNA substrate and cleaves the *N*-glycosidic bond between the deoxyribose and the hypoxanthine base, releasing the hypoxanthine base to form an apurinic/apyrimidinic site (AP site).^56^,^57^ Subsequently, the AP site in the dsDNA substrate can be catalyzed by apurinic/apyrimidinic endonuclease (APE1)^58^ to release 5′-biotin-labeled hAAG primer with free 3′-OH terminus (Fig. S1A, ESI†). The released hAAG primer (green color, Scheme 1) can pair with the hAAG circular template (grass green color, Scheme 1), initiating RCA reaction in the presence of phi29 polymerase and four kinds of deoxyribonucleotides (*i.e.*, dATP, dTTP, Cy3-dCTP and Cy5-dGTP). According to base matching rule, the hAAG circular template containing only three types of bases (*i.e.*, A, T, and G) may result in the hAAG amplification product containing only three types of bases (*i.e.*, T, A, and C), and the introduction of Cy3-modified dCTP leads to the incorporation of a large number of Cy3 fluorescent molecules in the RCA product. Similarly, when UDG is present, it can remove uracil base from DNA by catalyzing the hydrolysis of *N*-glycosidic bond between deoxyribose and uracil base for the generation of an abasic site. Subsequently, the AP site in the dsDNA substrate can be catalyzed by APE1 to release the 5′-biotin-labeled UDG primer with free 3′-OH terminus (red color, Scheme 1), which can pair with the UDG circular template (orange color, Scheme 1) to initiate RCA reaction (Fig. S1B, ESI†). Since the UDG circular template contains only three types of bases (*i.e.*, A, T and C), the introduction of Cy5-modified dGTP leads to the incorporation of a large number of Cy5 fluorescent molecules in the RCA product according to base matching rule. After magnetic separation, the amplification products of hAAG and UDG with biotin at the 5′ terminus are separated from the reaction solution and are subsequently digested into single nucleotides by Exonucleases I and III, releasing Cy3 and Cy5 fluorescent molecules. The Cy3 and Cy5 fluorescent molecules can be simply counted by TIRF-based single-molecule detection for the quantification of hAAG and UDG, respectively. The maximum emission wavelength is 568 nm for Cy3 and 670 nm for Cy5, without spectral overlap between the emission of Cy3 and that of Cy5 (Fig. S2, ESI†), and thus Cy3 signal and Cy5 signal can be used to indicate the presence of hAAG and UDG, respectively. In contrast, in the absence of DNA glycosylases, none base in the bifunctional dsDNA substrate can be removed and none primer is released. Neither RCA reaction nor the release of Cy3 and Cy5 fluorescent molecules into the solution can occur. Thus, neither Cy3 nor Cy5 signal can be detected.

Notably, with the introduction of RCA-based isothermal amplification and fluorescent-labeled nucleotide analogs (Cy3-dCTP and Cy5-dGTP), our method enables the controlled fluorescent labeling and signal amplification for the simultaneous detection of multiple DNA glycosylases. In this research, the involved RCA is an isothermally amplification^45^,^46^,^59^ with the capability of direct fluorescent labelling, and it produces biotin-/multiple Cy3-labeled ssDNA for hAAG and biotin-/multiple Cy5-labeled ssDNA for UDG with tandem repeats. The number of Cy3/Cy5 fluorescent molecules increase with the amounts of repeated sequences of RCA product and the complementary bases in circular template (*i.e.*, G bases for the hAAG circular template and C bases for the UDG circular template). In general, *n* repeats with *m* complementary bases can generate *nm* fluorescent molecules per RCA product. Since the hAAG circular template consists of 19 G bases and the UDG circular template consists of 19 C bases (*m* = 19), theoretically RCA allows for 19 fluorescent molecules per concatemer through the incorporation of Cy3-dCTP/Cy5-dGTP into the RCA product, and the incorporation number of fluorescent molecules in hAAG/UDG amplification product is estimated to be 19*n* Cy3 and 19*n* Cy5 molecules, respectively. Therefore, taking advantage of RCA-driven encoding of different fluorescent molecules and the high signal-to-noise ratio of single-molecule detection, the proposed method can be applied for sensitive detection of multiple DNA glycosylases.

### Validation of the assay

**Fig. 1:**
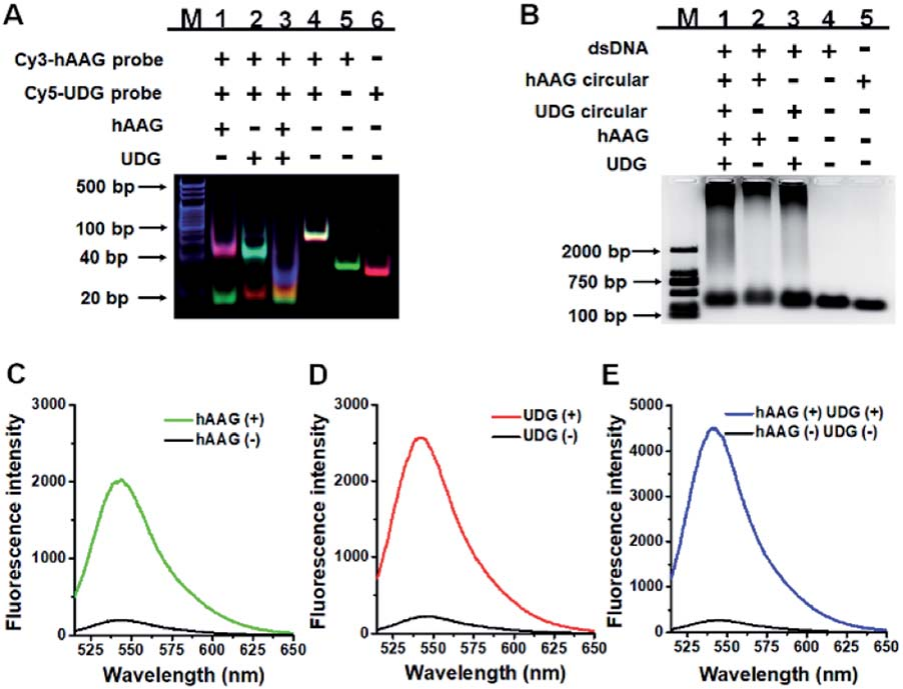
(A) Nondenaturating PAGE analysis of the DNA glycosylases-induced excision reaction products. Lane M: 20 bp DNA ladder marker; lane 1, Cy3-labeled hAAG probe + Cy5-labeled UDG probe + hAAG; lane 2, Cy3-labeled hAAG probe + Cy5-labeled UDG probe + UDG; lane 3, Cy3-labeled hAAG probe + Cy5-labeled UDG probe + hAAG + UDG; lane 4, Cy3-labeled hAAG probe + Cy5-labeled UDG probe; lane 5, Cy3-labeled hAAG probe; lane 6, Cy5-labeled UDG probe. (B) Agarose gel electrophoresis analysis of the DNA glycosylases-induced RCA products. Lane M: 2000 bp DNA ladder marker; lane 1, bifunctional dsDNA substrate (hybridization of hAAG probe with UDG probe) + hAAG circular template + UDG circular template + hAAG + UDG; lane 2, dsDNA substrate + hAAG circular template + hAAG; lane 3, dsDNA substrate + UDG circular template + UDG; lane 4, dsDNA substrate; lane 5, hAAG circular template. (C) Fluorescence measurement of the hAAG-induced RCA products in the presence of dsDNA substrate + hAAG circular template + hAAG (green line) and dsDNA substrate + hAAG circular template (black line). SYBR Gold is used as the fluorescent indicator. (D) Fluorescence measurement of the UDG-induced RCA products in the presence of dsDNA substrate + UDG circular template + UDG (red line) and dsDNA substrate + UDG circular template (black line). (E) Fluorescence measurement of the hAAG- and UDG-induced RCA products in the presence of dsDNA substrate + hAAG circular template + UDG circular template + hAAG + UDG (blue line) and dsDNA substrate + hAAG circular template + UDG circular template (black line). The concentration of each circular template is 50 nM. The 100 nM bifunctional dsDNA concentration, 0.1 U μL^–1^ hAAG and 0.1 U μL^–1^ UDG were used in this research.

We performed gel electrophoresis and fluorescence measurement to investigate the feasibility of the proposed method for hAAG and UDG assay (Fig. 1). We used non-denaturating polyacrylamide gel electrophoresis (PAGE) to verify the excision repair reaction (Fig. 1A). The hybridization of Cy3-labeled hAAG probe (Table 1 and Fig. 1A, lane 5) with Cy5-labeled UDG probe (Fig. 1A, lane 6) forms a bifunctional dsDNA substrate (Fig. 1A, lane 4), and thus the observed Cy3-labeled hAAG primer fragment can indicate the hAAG-actuated hypoxanthine excision repair reaction, and the observed Cy5-labeled UDG primer fragment can indicate the UDG-actuated uracil excision repair reaction. As shown in Fig. 1A, in the absence of two glycosylases, only a dsDNA band resulting from the bifunctional dsDNA substrate can be observed with the co-localization of SYBR Gold, Cy3 and Cy5 in single band (Fig. 1A, line 4, white color), indicating that the dsDNA is intact (SYBR Gold channel is shown in blue, Cy3 channel is shown in green, Cy5 channel is shown in red). In the presence of hAAG or UDG, the dsDNA is cleaved, generating a 21 nt Cy3-labeled hAAG primer (Fig. 1A, lane 1, green color) or Cy5-labeled UDG primer (Fig. 1A, lane 2, red color) and the remained dsDNA fragments (Fig. 1A, lane 1, magenta color or lane 2, cyan color), indicating that hAAG/UDG can recognize the I:T/U:A base pair and excise the hypoxanthine/uracil specifically with the assistance of APE1. When both hAAG and UDG are present, both the green and red bands can be observed (Fig. 1A, lane 3), indicating that hAAG and UDG can efficiently cleave the bifunctional dsDNA substrate without the interference from each other. These results demonstrate that this strategy is feasible for simultaneous detection of hAAG and UDG.

**Table 1:** Sequences of the oligonucleotides^*a*^

Note	Sequence (5′–3′)
hAAG probe	Biotin – AAC ATC CCT AAT TTC TCA CTA **I[combining low line]**GC TAG CTC AGT CAT ACA CT – NH_2_
UDG probe	Biotin – AAG ATG GGT AAT TAG AGT GTA **U[combining low line]**GA CTG AGC TAG CTT AGT GA – NH_2_
hAAG circular template	**TAG TGA GAA ATT AGG GAT GTT** AAG TAG GAT GTT GAG TAA AGT TGA AGA ATG GTG A
UDG circular template	**TAC ACT CTA ATT ACC CAT CTT** AAC TAC CAT CTT CAC TAT TCA ACT TCA ATC CTC A
Cy3-labeled hAAG probe	Cy3 – AAC ATC CCT AAT TTC TCA CTA **I[combining low line]**GC TAG CTC AGT CAT ACA CT
Cy5-labeled UDG probe	Cy5 – AAG ATG GGT AAT TAG AGT GTA **U[combining low line]**GA CTG AGC TAG CTT AGT GA

^*a*^The underlined bold letter “I” is deoxyinosine, and the underlined bold letter “U” represents deoxyuridine. In the circular templates, the hybridization region with primers are shown in bold.

We further used agarose gel electrophoresis (Fig. 1B) and fluorescence measurement (Fig. 1C–E) to verify the DNA glycosylases-induced RCA reaction. A single band appears in the presence of either bifunctional dsDNA substrate (Fig. 1B, lane 4) or the hAAG circular template (Fig. 1B, lane 5). It should be noted that because the length of UDG circular template and that of hAAG circular template are similar, the migration band of UDG circular template is similar to that of hAAG circular template (Fig. 1B, lane 5). In addition, the migration rate of circular DNA in agarose gel electrophoresis is slower than that of linear DNA with the same molecular weight because circular DNA has larger steric hindrance.^60^,^61^ In contrast, the distinct amplification bands with different molecular weights appear in the presence of hAAG circular template + hAAG (Fig. 1B, lane 2), UDG circular template + UDG (Fig. 1B, lane 3), and hAAG circular template + UDG circular template + hAAG + UDG (Fig. 1B, lane 1), indicating the occurrence of DNA glycosylases-induced RCA reaction. The results of agarose gel electrophoresis (Fig. 1B) are consistent with those of fluorescence measurements (Fig. 1C–E). As shown in Fig. 1C–E (black line), no significant fluorescence signals are detected in the presence of dsDNA substrate + circular templates. In contrast, in the presence of dsDNA substrate + hAAG circular template + hAAG, a high fluorescence signal (Fig. 1C, green line) is observed with SYBR Gold as the fluorescent indicator. Similarly, a high fluorescence signal (Fig. 1D, red line) is detected in the presence of dsDNA substrate + UDG circular template + UDG. Notably, the fluorescence signal in response to UDG is slightly higher than that in response to hAAG, consistent with the RCA results obtained using the synthetic hAAG/UDG primer (Fig. S3, ESI†). In the presence of dsDNA substrate + hAAG circular template + UDG circular template + hAAG + UDG, a much higher fluorescence signal is detected (Fig. 1E, blue line). These results demonstrate that the two circular templates used in this research do not interfere with each other during RCA amplification and the proposed method can be used for the simultaneous detection of multiple DNA glycosylases.

**Fig. 2:**
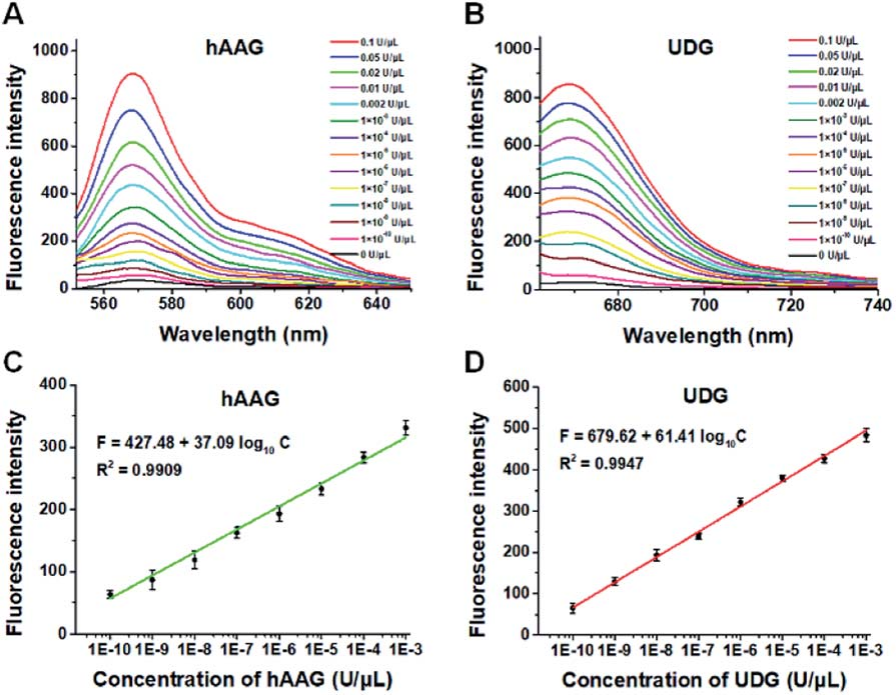
(A) Fluorescence spectra in response to different concentrations of hAAG. (B) Fluorescence spectra in response to different concentrations of UDG. (C) The log–linear correlation between the fluorescence intensity at 568 nm and the concentration of hAAG. (D) The log–linear correlation between the fluorescence intensity at 670 nm and the concentration of UDG. Error bars show the standard deviations of three experiments. The 100 nM bifunctional dsDNA substrates and 2 U of APE1 were used in this research.

To demonstrate the proof of concept, we monitored the change of Cy3 and Cy5 fluorescence intensities in response to various concentrations of hAAG and UDG, respectively. After the assembly of RCA products onto the streptavidin-coated magnetic beads (MBs), the hAAG-/UDG-triggered amplification products can efficiently bind to the MBs through specific biotin-streptavidin interaction (Figs. S4 and S5, ESI†). As shown in Figs. 2A and B, the fluorescence intensities at the emission wavelength of 568 nm and 670 nm enhance with the increasing concentrations of hAAG and UDG from 1 × 10^–10^ to 0.1 U μL^–1^, respectively, and the fluorescence intensities exhibit a linear correlation with the logarithm of hAAG and UDG concentrations over a large dynamic range of 7 orders of magnitude from 1 × 10^–10^ to 1 × 10^–3^ U μL^–1^ (Figs. 2C and D), respectively. The regression equations are *F* = 427.48 + 37.09 log_10_ *C* (*R*^2^ = 0.9909) for hAAG assay and *F* = 679.62 + 61.41 log_10_ *C* (*R*^2^ = 0.9947) for UDG assay, where *F* is the fluorescence intensity and *C* is the concentration of DNA glycosylases (U μL^–1^). The limit of detection (LOD) is calculated to be 8.69 × 10^–11^ U μL^–1^ for hAAG and 5.20 × 10^–11^ U μL^–1^ for UDG based on the evaluation of the average response of the control group plus three times the standard deviation. The sensitivity of hAAG has been improved by as much as 6 orders of magnitude compared with that of magnetic bead-based fluorescent assay (1 × 10^–4^ U μL^–1^)^25^ and hyperbranched signal amplification-based fluorescent assay (9 × 10^–5^ U μL^–1^),^62^ and 5 order of magnitude compared with that of base excision repair-mediated cascading triple-signal amplification (2.6 × 10^–5^ U μL^–1^).^63^ The sensitivity of UDG has been improved by 5 orders of magnitude compared with that of luminescent assay (2 × 10^–5^ U μL^–1^),^64^ 3 order of magnitude compared with that of enzyme-assisted bicyclic cascade signal amplification-based fluorescent assay (1 × 10^–7^ U μL^–1^),^23^ and at least 2 orders of magnitude compared with those of RCA-based assays (1.4 × 10^–7^ U μL^–1^ to 1.7 × 10^–8^ U μL^–1^).^30^–^34^

### Single-molecule detection of multiple DNA glycosylases

**Fig. 3:**
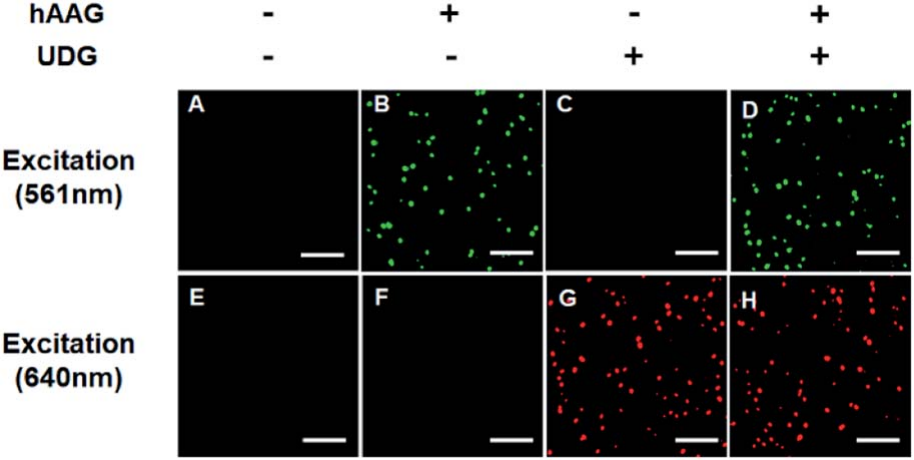
Simultaneous detection of hAAG and UDG by TIRF-based single-molecule imaging. The Cy3 fluorescence signal is shown in green, and the Cy5 fluorescence signal is shown in red. The 1 × 10^–3^ U μL^–1^ hAAG, 1 × 10^–3^ U μL^–1^ UDG, 100 nM bifunctional dsDNA substrates and 2 U of APE1 were used in this research. The scale bar is 5 μm.

The fluorescence signals of Cy3 and Cy5 were detected simultaneously by TIRF microscopy, with Cy3 indicating the presence of hAAG and Cy5 indicating the presence of UDG. In the absence of hAAG and UDG (*i.e.*, with only APE1 present), neither Cy3 fluorescence signal (Fig. 3A) nor Cy5 fluorescence signal (Fig. 3E) can be detected. In the presence of hAAG, distinct Cy3 fluorescence signals are detected (Fig. 3B), but no Cy5 fluorescence signal is observed (Fig. 3F). While in the presence of UDG, distinct Cy5 fluorescence signals are detected (Fig. 3G), but no significant Cy3 fluorescence signal is observed (Fig. 3C). Only in the presence of both hAAG and UDG, can Cy3 (Fig. 3D) and Cy5 (Fig. 3H) fluorescence signals be observed simultaneously. These results clearly demonstrate that the proposed method can be used for the simultaneous detection of multiple DNA glycosylases at the single-molecule level. Notably, APE1 is necessary for the cleavage of AP sites by hAAG and UDG (Fig. S6, ESI†), and this method can even detect low-concentration hAAG (1 × 10^–11^ U μL^–1^) and UDG (1 × 10^–11^ U μL^–1^) in the presence of APE1 (Fig. S7, ESI†).

**Fig. 4:**
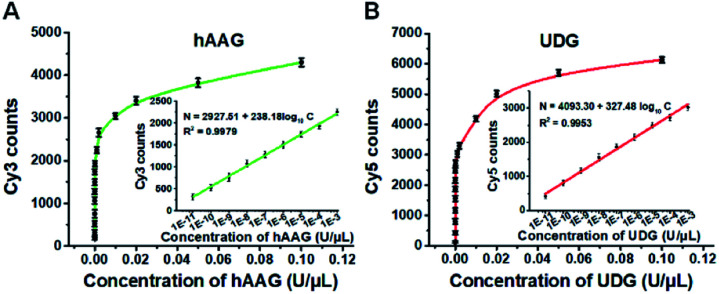
(A) Variance of Cy3 counts with the hAAG concentration. The inset shows the linear relationship between Cy3 counts and the logarithm of hAAG concentration in the range from 1 × 10^–11^ to 1 × 10^–3^ U μL^–1^. (B) Variance of Cy5 counts with the UDG concentration. The inset shows the linear relationship between Cy5 counts and the logarithm of UDG concentration in the range from 1 × 10^–11^ to 1 × 10^–3^ U μL^–1^. The 100 nM bifunctional dsDNA substrates and 2 U of APE1 were used in this research. Error bars show the standard deviation of three experiments.

To investigate the detection sensitivity of the proposed method, we further monitor the variance of Cy3 and Cy5 counts with different concentrations of hAAG and UDG under the optimally experimental conditions (Fig. S8–S12, ESI†), respectively. As shown in Fig. 4A and B, the Cy3 and Cy5 counts improve with the increasing concentrations of hAAG and UDG from 1 × 10^–11^ to 0.1 U μL^–1^, respectively. In the logarithmic scale, the counts of Cy3 and Cy5 exhibit a linear correlation with the concentrations of hAAG and UDG over 8 orders of magnitude from 1 × 10^–11^ to 1 × 10^–3^ U μL^–1^ (insets of Fig. 4A and B), respectively. The regression equations are *N* = 2927.51 + 238.18 log_10_ *C* (*R*^2^ = 0.9979) for hAAG assay and *N* = 4093.30 + 327.48 log_10_ *C* (*R*^2^ = 0.9953) for UDG assay, where *N* is the Cy3/Cy5 counts and *C* is the concentration of hAAG/UDG (U μL^–1^). The detection limit is calculated to be 6.10 × 10^–12^ U μL^–1^ for hAAG and 1.54 × 10^–12^ U μL^–1^ for UDG, respectively. Notably, the sensitivity of our single-molecule detection method has been improved by 14.25-fold and 33.77-fold compared with that of ensemble fluorescence measurement for hAAG and UDG assays, respectively (Fig. 2). The improved sensitivity might be ascribed to (1) the RCA-driven incorporation of large amounts of fluorescent molecules per concatemer, and (2) the high signal-to-noise ratio of single-molecule detection.

### Detection selectivity

**Fig. 5:**
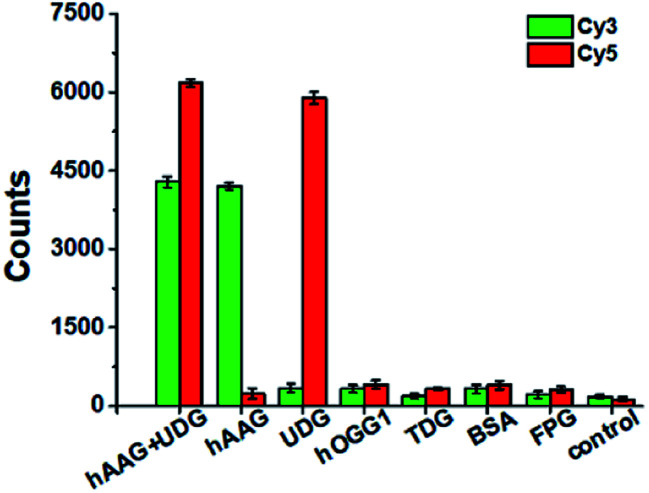
Variance of Cy3 counts (green color) and Cy5 counts (red color) in response to 0.1 U μL^–1^ hAAG + 0.1 U μL^–1^ UDG, 0.1 U μL^–1^ hAAG, 0.1 U μL^–1^ UDG, 0.1 U μL^–1^ hOGG1, 0.1 U μL^–1^ TDG, 0.1 μg μL^–1^ BSA, 0.2 U μL^–1^ FPG, and the control group with only reaction buffer, respectively. The 100 nM bifunctional dsDNA substrates and 2 U of APE1 were used in this research. Error bars show the standard deviation of three experiments.

To evaluate the selectivity of the proposed method, we used human 8-oxoguanine-DNA glycosylase 1 (hOGG1), thymine DNA glycosylase (TDG), bovine serum albumin (BSA), and formamidopyrimidine [fapy]-DNA glycosylase (FPG) as the interference enzymes. The hOGG1 is 8-oxoguanine-specific glycosylase responsible for the oxidized guanine repair through the BER system.^65^ The TDG can selectively remove T from the G/T mismatches.^66^ BSA cannot recognize and excise the damaged bases from DNA substrate.^23^ FPG catalyzes the release of imidazole ring-opened forms of guanine and adenine from the alkylated/irradiated polynucleotides.^67^ In theory, none of these enzymes is able to recognize and cleave the bifunctional dsDNA substrates to generate the primers, and thus there is no subsequent RCA reaction. As shown in Fig. 5, neither Cy3 nor Cy5 fluorescence signal is detected in the presence of hOGG1, TDG, BSA and FPG. In contrast, when both hAAG and UDG are present, Cy3 and Cy5 fluorescence signals can be simultaneously observed. In the presence of hAAG, only a high Cy3 fluorescence signal is observed. While in the presence of UDG, only a high Cy5 fluorescence signal is observed. These results demonstrate that the proposed method possesses good specificity towards hAAG and UDG.

### Kinetic analysis

**Fig. 6:**
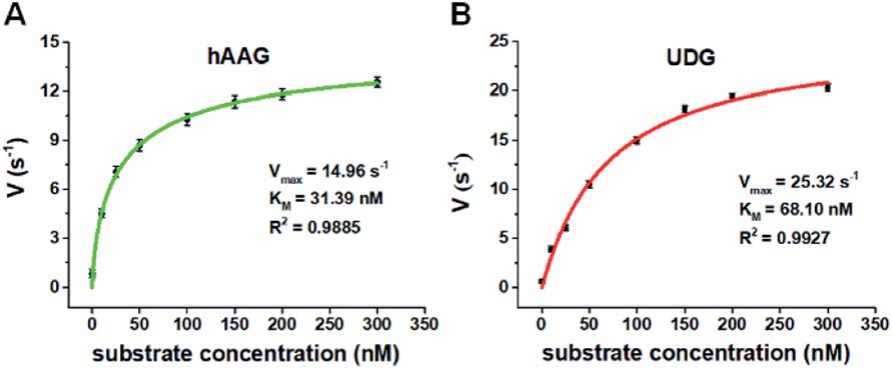
(A) Variance of initial velocity (*V*) with the concentration of DNA substrates in response to 0.1 U μL^–1^ hAAG. (B) Variance of initial velocity (*V*) with the concentration of DNA substrates in response to 0.1 U μL^–1^ UDG. Error bars show the standard deviation of three experiments.

We further applied this method to quantify the kinetic parameters at the single-molecule level. The initial velocities (*V*) are determined in the presence of 0.1 U μL^–1^ hAAG and 0.1 U μL^–1^ UDG, respectively, with variable-concentration of DNA substrate between 0 and 300 nM in 5 min reaction at 37 °C.^68^ As shown in Fig. 6, the initial velocities of both hAAG (Fig. 6A) and UDG (Fig. 6B) enhance with the increasing concentrations of DNA substrates. The experimental data are fitted to the Michaelis–Menten equation *V* = *V*_max_[S]/(*K*_m_ + [S]), where *V*_max_ is the maximum initial velocity, [S] is the concentration of DNA substrates, and *K*_m_ is the Michaelis–Menten constant corresponding to the concentration at half-maximal velocity.^69^ The *V*_max_ of hAAG is evaluated to be 14.96 s^–1^ and *K*_m_ is calculated to be 31.39 nM, consistent with that obtained by the radioactive assay (13–42 nM).^70^ The *V*_max_ of UDG is determined to be 25.32 s^–1^ and *K*_m_ is calculated to be 68.10 nM, consistent with that obtained by molecular beacon-based fluorescent method (60 nM).^71^ These results demonstrate that the proposed method can be used to accurately evaluate the kinetic parameters of multiple DNA glycosylases.

### Inhibition assay

**Fig. 7:**
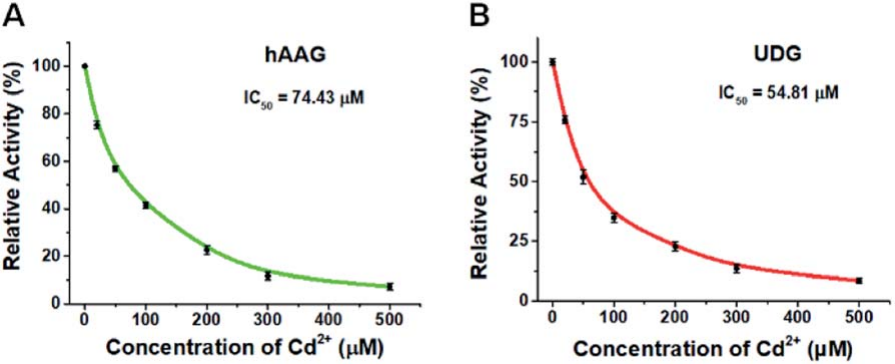
(A) Variance of the relative activity of hAAG in response to different-concentration Cd^2+^. (B) Variance of the relative activity of UDG in response to different-concentration Cd^2+^. The 100 nM bifunctional dsDNA substrates, 2 U of APE1, 0.1 U μL^–1^ hAAG, and 0.1 U μL^–1^ UDG were used in this research. Error bars show the standard deviation of three experiments.

To demonstrate the capability of the proposed method for DNA glycosylases inhibition assay, we used cadmium (Cd^2+^) as the model inhibitor.^72^,^73^ As shown in Fig. 7, the relative activities of hAAG and UDG decrease with the increasing concentration of Cd^2+^, respectively. Based on the plot of relative activity of hAAG *versus* Cd^2+^ concentration (Fig. 7A), the half-maximal inhibitory concentration (IC_50_) value of hAAG in the presence of APE1 is calculated to be 74.43 μM, which is smaller than the value of hAAG alone measured by the radioactive assay (120 μM).^74^ This can be explained by the fact that Cd^2+^ inhibits not only the activity of hAAG but also the activity of APE1 in the range of 10–100 μM.^75^ Similarly, Cd^2+^ can effectively inhibit UDG with an IC_50_ value of 54.81 μM in the presence of APE1 (Fig. 7B). Because Cd^2+^ may lead to the inactivation of UDG and APE1,^75^ the obtained IC_50_ is smaller than the value of UDG alone measured by gel electrophoresis assay (70 μM).^72^ These results clearly demonstrate that the proposed method can be applied to simultaneously screen the inhibitors of hAAG and UDG.

### Detection of cellular DNA glycosylases

**Fig. 8:**
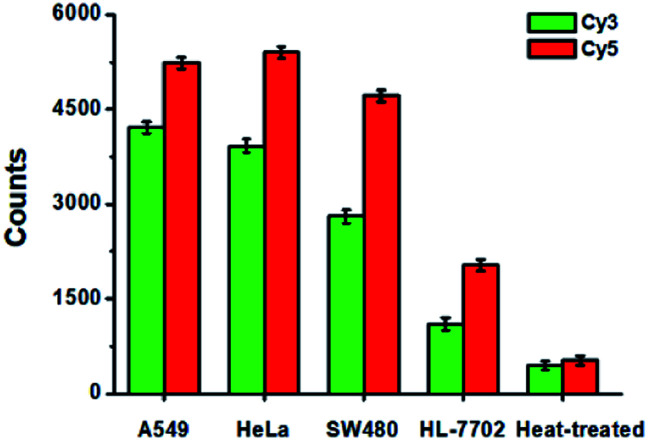
Measurement of Cy3 counts (green column) and Cy5 counts (red column) in response to the cell extracts (equivalent to 1000 cells) obtained from A549 cells, HeLa cells, SW480 cells, HL-7702 cells, and the heat-treated cell extracts, respectively. Error bars show the standard deviations of three experiments.

To evaluate the feasibility of the proposed method for clinical diagnosis, we further measured the activities of DNA glycosylases in different cell lines including the human lung adenocarcinoma cell line (A549 cells), human cervical carcinoma cell line (HeLa cells), human colon cancer cells (SW480 cells), human hepatocyte cell line (HL-7702 cells), and the heat-inactivated A549 cell extracts. As shown in Fig. 8, high Cy5 counts and Cy3 counts are detected in the presence of A549 cells, HeLa cells, and SW480 cells, respectively, consistent with the over-expression of glycosylases in human cancer cells.^14^,^15^ However, no distinct Cy3 and Cy5 counts are detected in the presence of HL-7702 cells due to the low activities of glycosylases in normal cells. Notably, when A549 cell extracts are heat-treated, no distinct Cy3 and Cy5 counts are observed due to the loss of glycosylases activity.

**Fig. 9:**
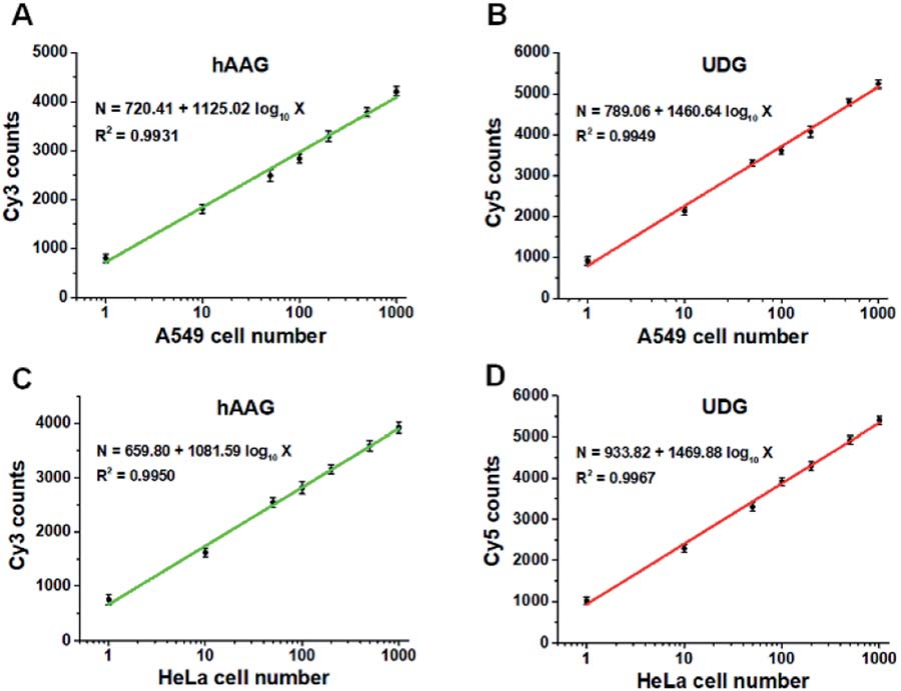
(A and B) Measurement of Cy3 counts (A) and Cy5 counts (B) in the presence of A549 cells, respectively. (C and D) Measurement of Cy3 counts (C) and Cy5 counts (D) in the presence of HeLa cells, respectively. The 100 nM bifunctional dsDNA substrates and 2 U of APE1 were used in this research. Error bars represent standard deviations of three experiments.

Fig. 9A and B show the variance of Cy3 counts and Cy5 counts as a function of the number of A549 cells. Notably, the counts of Cy3 and Cy5 exhibit linear correlation with the logarithm of A549 cell number in the range from 1 to 1000 cells, respectively. The correlation equations are *N* = 720.41 + 1125.02 log_10_ *X* (*R*^2^ = 0.9931) for hAAG assay (Fig. 9A) and *N* = 789.06 + 1460.64 log_10_ *X* (*R*^2^ = 0.9949) for UDG assay (Fig. 9B), where *X* is the number of A549 cells and *N* is the counts of fluorescent molecules (Cy3 for hAAG assay and Cy5 for UDG assay). The limit of detection is calculated to be 1 cell for both hAAG assay and UDG assay. We further investigated the variance of Cy3 counts and Cy5 counts as a function of the number of HeLa cells (Fig. 9C and D). The Cy3 counts (Fig. 9C) and Cy5 counts (Fig. 9D) enhance with the increasing number of HeLa cells, and a good linear correlation is obtained between the counts of fluorescent molecules and the logarithm of HeLa cell number in the range of 1 to 1000. The correlation equations are *N* = 659.80 + 1081.59 log_10_ *X* (*R*^2^ = 0.9950) for hAAG assay (Fig. 9C) and *N* = 933.82 + 1469.88 log_10_ *X* (*R*^2^ = 0.9967) for UDG assay (Fig. 9D), where *X* represents the number of HeLa cells and *N* represents the counts of fluorescent molecules (Cy3 for hAAG assay and Cy5 for UDG assay). The limit of detection is calculated to be 1 cell for both hAAG assay and UDG assay. These results clearly demonstrate that the proposed method can be applied for quantitative detection of multiple DNA glycosylases even at the single-cell level, holding great potential for further application in clinical diagnosis.

## Conclusions

In summary, we have developed a sensitive method for simultaneous detection of multiple DNA glycosylases based on the integration of single-molecule detection with RCA-driven encoding of different fluorescent molecules. This method has significant advantages of high amplification efficiency, high throughput, and easy design. (1) This strategy can greatly increase the number of fluorescent molecules per concatemer through RCA-driven encoding of different fluorescent molecules. (2) In contrast to SYBR Green I which can only detect a single target,^34^ this strategy enables the simultaneous detection of multiple DNA glycosylases. (3) The circular templates involved in this assay can be easily designed.^32^ In contrast to the involvement of sophisticated detection probe design (*e.g.*, fluorescently labelled oligonucleotide probe modified with a fluorophore and a quencher) for hAAG/UDG assays,^23^,^25^,^32^,^62^,^63^ our method enables the simultaneous detection of multiple DNA glycosylases through the introduction of RCA-driven encoding of different fluorescent molecules, without the requirement of specially labelled detection probes for simultaneous detection. Owing to the high amplification efficiency of RCA and the high signal-to-noise ratio of single-molecule detection, this method is extremely sensitive with a detection limit of 6.10 × 10^–9^ U mL^–1^ for hAAG and 1.54 × 10^–9^ U mL^–1^ for UDG, superior to the reported magnetic bead-based fluorescent assay^25^ and the luminescent assay.^64^ This method can discriminate normal cells from cancer cells with high expression of DNA glycosylases, and it can be further applied for the simultaneous detection of multiple DNA glycosylases in cancer cells at the single-cell level and the screening of DNA glycosylase inhibitors. Importantly, by simply changing the recognition sites in the bifunctional DNA substrate, this method can be extended to simultaneously detect other kinds of multiple enzymes, holding great potential in biomedical research, clinical diagnosis, and drug discovery.

## Experimental

### Materials

All oligonucleotides (Table 1) were synthesized by TaKaRa Biotechnology Co., Lid. (Dalian, China). Two circular templates were prepared from the corresponding linear templates by TaKaRa Biotechnology Co., Lid. (Dalian, China). The human alkyladenine DNA glycosylase (hAAG, 10 U μL^–1^, 3.88 μM), *E. coli* uracil–DNA glycosylase (UDG, 5 U μL^–1^, 1.72 μM), human apurinic/apyrimidinic endonuclease 1 (APE1), phi29 DNA polymerase, Exonuclease I (Exo I), Exonuclease III (Exo III), human 8-oxoguanine-DNA glycosylase 1 (hOGG1), formamidopyrimidine [fapy]-DNA glycosylase (FPG), bovine serum albumin (BSA), 10× NEBuffer 4 (500 mM potassium acetate, 200 mM Tris-acetate, 100 mM magnesium acetate, 10 mM DTT, pH 7.9), 10× UDG reaction buffer (200 mM Tris–HCl, 10 mM DTT, 10 mM EDTA, pH 8), 10× phi29 DNA polymerase reaction buffer (500 mM Tris–HCl, 100 mM MgCl_2_, 100 mM (NH_4_)_2_SO_4_, 40 mM DTT, pH 7.5), 10× NEBuffer 1 (10 mM bis-tris-propane–HCl, 10 mM MgCl_2_, 1 mM DTT, pH 7.0), and 10× Exonuclease I reaction buffer (670 mM glycine–KOH, 67 mM MgCl_2_, 100 mM β-ME, pH 9.5) were purchased from New England Biolabs (Ipswich, MA, USA). Thymine DNA glycosylase (TDG) was obtained from R&D System (Minneapolis, MN, USA). The dATP and dTTP were purchased from TaKaRa Biotechnology Co. Ltd. (Dalian, China). Cyanine 3-dCTP (Cy3-dCTP) and Cyanine 5-dGTP (Cy5-dGTP) were acquired from PerkinElmer (Foster City, CA, USA). The streptavidin-coated magnetic beads (MBs) and SYBR Gold were obtained from Invitrogen (California, CA, USA). Human lung adenocarcinoma cell line (A549 cells), human cervical carcinoma cell line (HeLa cells), human colon cancer cells (SW480 cells) and human hepatocyte cell line (HL-7702 cells) were purchased from Cell Bank of Chinese Academy of Sciences (Shanghai, China). Nuclear extract kit was brought from Active Motif (Carlsbad, CA, USA). All other reagents were of analytical grade and used just as received without further purification. The ultrapure water used in the experiments was prepared by Millipore filtration system (Millipore, Milford, MA, USA).

### Preparation of bifunctional dsDNA substrates

The 1 μM hAAG probe and 1 μM UDG probe were incubated in an annealed buffer containing 10 mM Tris–HCl (pH 8.0), 50 mM NaCl, and 1 mM EDTA at 95 °C for 5 min, followed by slowly cooling to room temperature to form the bifunctional dsDNA substrates. The obtained bifunctional dsDNA substrates were stored at 4 °C for further use.

### DNA glycosylases-induced excision reaction and RCA reaction

The DNA glycosylases-induced excision reaction was performed in 10 μL of reaction solution containing 100 nM bifunctional dsDNA substrates, 1× NEBuffer 4, 1× UDG reaction buffer, 2 U of APE1, and different concentrations of hAAG and UDG at 37 °C for 1 h. Then 50 nM hAAG circular template, 50 nM UDG circular template, 0.1 mg mL^–1^ BSA, 0.25 mM dATP, 0.25 mM dTTP, 10 μM Cy3-dCTP, 10 μM Cy5-dGTP, 1× phi29 reaction buffer, and 5 U of phi29 polymerase were added to the reaction solution with a final volume of 20 μL. Subsequently, the solution was incubated at 30 °C in the dark for 2 h, and the reaction was terminated by incubation at 65 °C for 10 min.

### Conjugation of amplification products with the streptavidin-coated magnetic beads

The 20 μL of biotinylated amplification product was mixed with 10 μL of 5 μg μL^–1^ streptavidin-coated MBs solution, and incubated in the dark for 15 min on a roller mixer at room temperature. Then the mixture was washed three times by magnetic separation using 1× binding and washing buffer (5 mM Tris–HCl (pH 7.5), 0.5 mM EDTA, 1 M NaCl) to remove the excess Cy3-dCTP and Cy5-dGTP, and the ssDNAs-MB conjugates (MB-ssDNAs) were resuspended in 1× NEBuffer 1.

### Exonuclease digestive reaction

The exonuclease digestive reaction was performed in 20 μL of reaction mixture containing the MB-ssDNAs, 10 U of Exo III, 10 U of Exo I, 1× NEBuffer 1, and 1× Exo I reaction buffer at 37 °C for 30 min. Then the streptavidin-coated MBs were separated by magnetic separation for 3 min in the darkness, and the supernatant solution was subjected to measurements.

In the ensemble fluorescence measurement, 20 μL of reaction products was diluted to a final volume of 80 μL, and then subjected to the fluorescence spectra measurement by using Hitachi F-7000 spectrometer (Tokyo, Japan) equipped with a xenon lamp as the excitation source. The Cy3 fluorescence spectra were measured at an excitation wavelength of 532 nm, and the fluorescence intensity at 568 nm was used for quantitative analysis of hAAG. The Cy5 fluorescence spectra were measured at an excitation wavelength of 635 nm, and the fluorescence intensity at 670 nm was used for quantitative analysis of UDG.

### Single-molecule detection and data analysis

In the single-molecule measurement, the reaction products were diluted 200-fold with the imaging buffer (3 mM MgCl_2_, 100 mM Tris–HCl (pH 8.0), 10 mM (NH_4_)_2_SO_4_). The 10 μL of sample was spread on a glass coverslip for imaging. The images of single molecules were acquired by total internal reflection fluorescence (TIRF) microscopy (Nikon, Ti-E, Japan). The 561 nm and 640 nm lasers were used to excite Cy3 and Cy5 fluorescent molecules, respectively. The photons of Cy3 and Cy5 were collected by an oil immersion 100× objective, and were split up into Cy3 channel (573–613 nm filter) and Cy5 channel (661.5–690.5 nm filter) by the dichroic mirror, and were imaged onto an EMCCD camera (Photometrics, Evolve 512). For data analysis, regions of interest of 600 × 600 pixels were selected for Cy3 and Cy5 fluorescent molecule counting by using ImageJ software. The numbers of Cy3 and Cy5 fluorescent molecules were the sum of ten frames, respectively.

### Gel electrophoresis

The DNA glycosylase reaction products were analyzed by 12% nondenaturing polyacrylamide gel electrophoresis (PAGE) in TBE buffer (44.5 mM Tris–boric acid, 1 mM EDTA, pH 8.2) at a 110 V constant voltage for 50 min at room temperature. Then the gel was stained with SYBR gold and analyzed by Bio-Rad ChemiDoc MP Imaging System (Hercules, CA, USA). The fluorescent DNA fragments of the enzyme reaction products were analyzed by using an illumination source of Epi-blue (460–490 nm excitation) and a 516–544 nm filter for SYBR Gold fluorophores, an illumination source of Epi-green (520–545 nm excitation) and a 577–613 nm filter for the Cy3 fluorophores, and an illumination source of Epi-red (625–650 nm excitation) and a 675–725 nm filter for Cy5 fluorophores. The RCA reaction products stained with SYBR Gold were analyzed by 1% agarose gel electrophoresis in TAE buffer (40 mM Tris-acetic acid, 2 mM EDTA) at a 110 V constant voltage for 60 min.

### Inhibition assay

For DNA glycosylases inhibition assay, various concentrations of CdCl_2_ were incubated with 1 U of hAAG, 1 U of UDG and 2 U of APE1 in DNA glycosylase reaction buffer at 37 °C for 15 min. Subsequently, DNA glycosylases-induced excision reaction, rolling circle amplification and exonuclease digestive reaction were performed as described above. The relative activity of DNA glycosylase (RA) was measured according to eqn (1):1
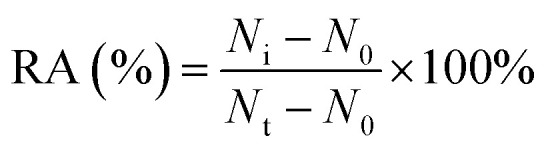
where *N*_0_ is the counts of fluorescent molecules when DNA glycosylase is absent, *N*_t_ is the counts of fluorescent molecules when DNA glycosylase is present, and *N*_i_ is the counts of fluorescent molecules in the presence of both DNA glycosylase and CdCl_2_. The IC_50_ value was calculated from the curve of RA *versus* the CdCl_2_ concentration.

### Cell culture and preparation of cell extracts

A549 cells, HeLa cells, SW480 cells and HL-7702 cells were cultured with 10% fetal bovine serum (FBS, Gibco, USA) and 1% penicillin-streptomycin (Invitrogen, USA) in Dulbecco's modified Eagle's medium (DMEM). The cells were cultured at 37 °C in a humidified atmosphere containing 5% CO_2_. The nuclear extracts were prepared by using the nuclear extract kit (ActiveMotif, Carlsbad, CA, USA) according to the manufacturer's protocol. The obtained supernatant fraction was subjected to hAAG and UDG assay.

## Conflicts of interest

There are no conflicts to declare.

## Supplementary Material

Supplementary information
