# Supplementary material for: Rolling circle amplification-driven encoding of different fluorescent molecules for simultaneous detection of multiple DNA repair enzymes at the single-molecule level
Source: Chem Sci. 2020 May 18;11(22):5724–34. doi: 10.1039/d0sc01652g (PMC7433776; doi:10.1039/d0sc01652g)
Supplement: Supplementary file 1 [file SC-011-D0SC01652G-s001.pdf]

Electronic Supplementary Material (ESI) for Chemical Science.
This journal is © The Royal Society of Chemistry 2020

Electronic Supplementary Information

Rolling circle amplification-driven encoding of different fluorescent molecules for simultaneous detection of multiple DNA repair enzymes at the single-molecule level

Chen-chen Li,‡ Hui-yan Chen,‡ Juan Hu* and Chun-yang Zhang*

College of Chemistry, Chemical Engineering and Materials Science, Collaborative Innovation Center of Functionalized Probes for Chemical Imaging in Universities of Shandong, Key Laboratory of Molecular and Nano Probes, Ministry of Education, Shandong Provincial Key Laboratory of Clean Production of Fine Chemicals, Shandong Normal University, Jinan 250014, China.

* Correspondence should be addressed to cyzhang@sdnu.edu.cn; juanhu@sdnu.edu.cn.

‡ These authors contributed equally.

Molecular mechanism of DNA glycosylases-mediated cleavage of bifunctional dsDNA substrate.

The hAAG is a mono-functional glycosylase specific for deoxyinosine base paired with a thymine (Fig. S1A).[1,2] As shown in Fig. S1A, hAAG specifically binds to DNA containing a target damaged base by inserting a β-hairpin loop into the minor groove of DNA. Glu125 acts as a general base to deprotonate a water molecule, which may serve as a nucleophile to attack the anomeric C1' carbon in an S_N 2 catalytic mechanism. In the presence of hAAG, the products are hypoxanthine and AP site. Subsequently, APE1 performs an incision at the 5' side of AP site, resulting in a nick in the phosphodiester backbone with 3'-OH and 5'-deoxyribose-5-phosphate (dRP) ends. Consequently, in the presence of hAAG, the bifunctional dsDNA substrate is selectively cleaved into two portions to produce hAAG-primer due to the combined glycosylase activity of hAAG and endonuclease activity of APE1.

The UDG is also a mono-functional glycosylase specific for uracil base paired with a thymine (Fig. S1B). As shown in Fig. S1B, UDG specifically binds to DNA containing a target damaged base by inserting a β-hairpin loop into the minor groove of DNA. Asp145 acts as a general base to deprotonate a water molecule, which may serve as a nucleophile to attack the anomeric C1' carbon in an S_N 2 catalytic mechanism. In the presence of UDG, the

products are deoxyuridine and AP site. Subsequently, APE1 performs an incision at the 5’ side of AP site, resulting in a nick in the phosphodiester backbone with 3’-OH and 5’-deoxyribose-5-phosphate (dRP) ends. Consequently, in the presence of UDG, the bifunctional dsDNA substrate is selectively cleaved into two portions to produce UDG-primer due to the combined glycosylase activity of UDG and endonuclease activity of APE1.

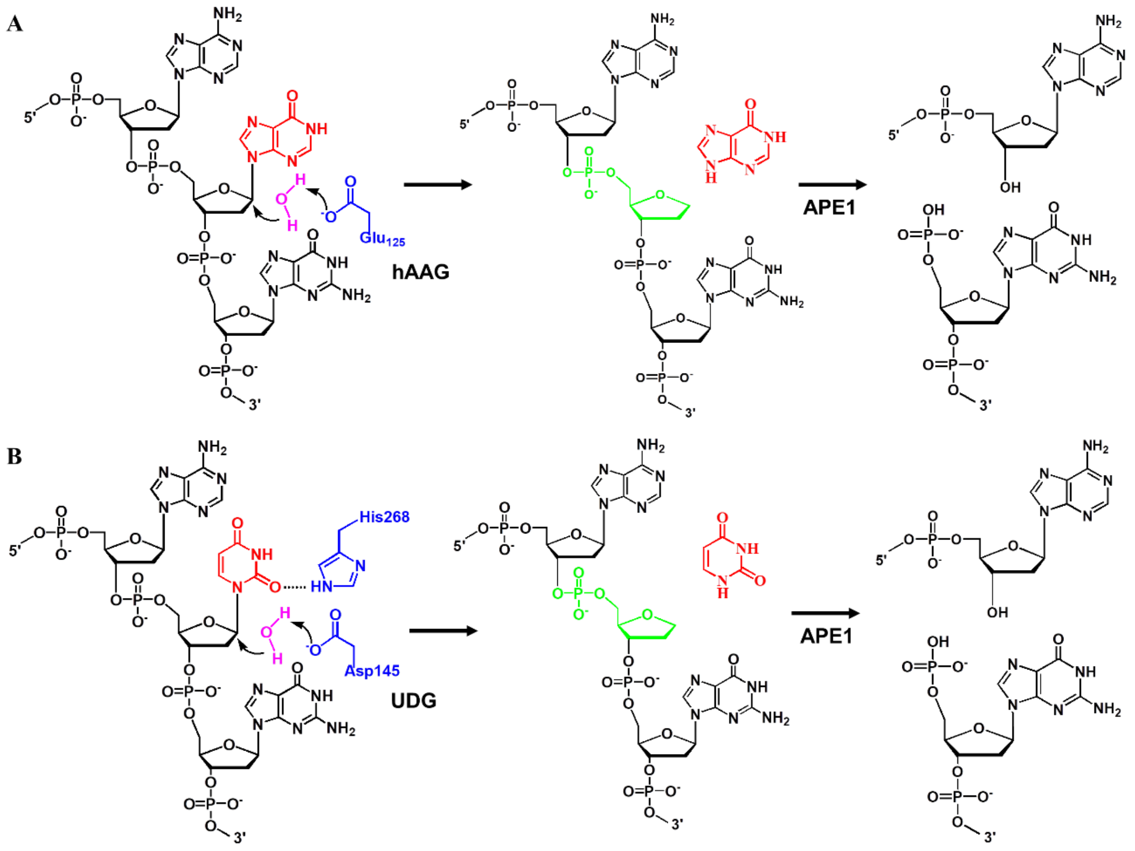

Fig. S1 (A) Mechanism of hAAG-mediated cleavage of bifunctional dsDNA substrate in the presence of APE1. The chemical steps of catalysis include step 1: hypoxanthine (Hx) base removal and formation of AP site, step 2: cleavage of the phosphodiester bond on the 5’ side at AP site. (B) Mechanism of UDG-mediated cleavage of bifunctional dsDNA substrate in the presence of APE1. The chemical steps of catalysis include step 1: uracil base removal and formation of AP site, step 2: cleavage of the phosphodiester bond on the 5’ side at AP site.

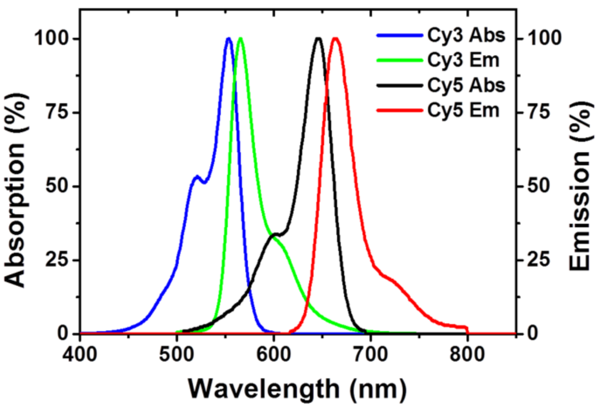

Fig. S2 Absorption and emission spectra of Cy3 and Cy5. Blue line: absorption spectrum of Cy3. Green line: emission spectrum of Cy3. Black line: absorption spectrum of Cy5. Red line: emission spectrum of Cy5.

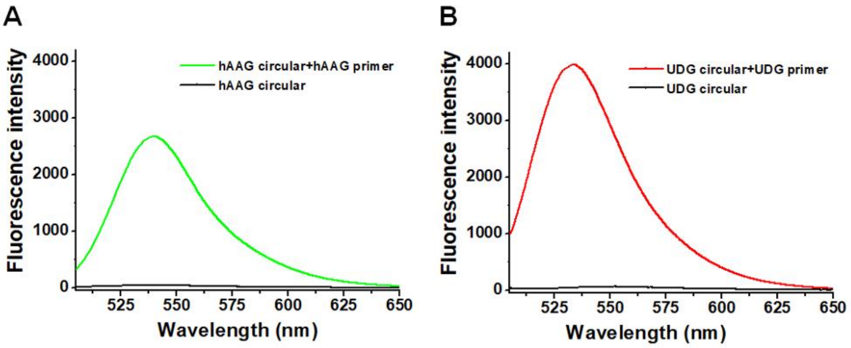

Fig. S3 (A) Fluorescence measurement of RCA products in the presence of hAAG circular template + synthetic hAAG primer (green line) and hAAG circular template (black line), respectively. The sequence of hAAG primer is 5'-biotin-AAC ATC CCT AAT TTC TCA CTA-OH-3'. (B) Fluorescence measurement of RCA products in the presence of UDG circular template + synthetic UDG primer (red line) and UDG circular template (black line), respectively. The sequence of UDG primer is 5'-biotin-AAG ATG GGT AAT TAG AGT GTA-OH-3'. SYBR Gold is used as the fluorescent indicator. The concentration of each circular template is 50 nM. The concentration of each primer is 100 nM. In the presence of the hAAG / UDG circular template (black lines), only low fluorescence signals are observed. In contrast, the enhanced fluorescence signals are detected in the presence of the hAAG circular template + hAAG primer (green line) and UDG circular template + UDG primer (red line), respectively, when the RCA products are stained with SYBR Gold. Moreover, the fluorescence signal in response to UDG primer is slightly higher than that in response to hAAG primer, consistent with the result of DNA

glycosylases-induced RCA (Higher fluorescence signal of UDG-induced RCA is observed) (Figs. 1C and 1D).

Study the conjugation of amplification products with the streptavidin-coated magnetic beads. The biotinylated amplification products were incubated with the streptavidin-coated MBs in the dark for 15 min on a roller mixer at room temperature. Then the mixture was washed three times by magnetic separation using 1× binding and washing buffer (5 mM Tris-HCl (pH 7.5), 0.5 mM EDTA, 1 M NaCl) to remove the excess Cy3-dCTP and Cy5-dGTP.

We measured the fluorescence emission spectra of Cy3 and Cy5 in the third washing supernatant in the presence of dsDNA substrate + APE1 + hAAG / UDG. As show in Fig. S4A, no significant Cy3 fluorescence signal is observed in the third washing supernatant (blue line), indicating that excess Cy3 fluorescent molecules have been removed. In contrast, an enhanced Cy3 fluorescence signal from the streptavidin-coated MBs (green line) is detected, which results from the hAAG-induced RCA product attached to the MBs. Similarly, no significant Cy5 fluorescence signal is observed in the third washing supernatant (Fig. S4B, cyan line), but an enhanced Cy5 fluorescence signal from the streptavidin-coated MBs (red line) is detected, which results from the UDG-induced RCA product attached to the MBs.

In the control groups with the presence of dsDNA substrate, dsDNA substrate + APE1, dsDNA substrate + hAAG, and dsDNA substrate + UDG, respectively, no significant fluorescence signal is observed (Fig. S5). In contrast, the enhanced fluorescence signals are detected in the presence of dsDNA substrate + APE1 + hAAG and dsDNA substrate + APE1 + UDG, respectively, as a result of hAAG- and UDG-triggered RCA products. These results demonstrate that the target glycosylase-triggered RCA products can efficiently bind to the MBs.

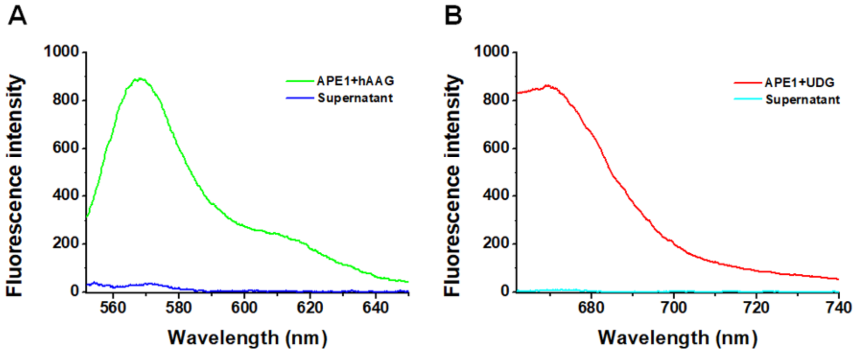

Fig. S4 (A) Measurement of Cy3 fluorescence emission spectra from the streptavidin-coated MBs (green line) and the third washing supernatant (blue line) in the presence of dsDNA substrate + APE1 + hAAG. (B) Measurement of

Cy5 fluorescence emission spectra from the streptavidin-coated MBs (red line) and the third washing supernatant (cyan line) in the presence of dsDNA substrate + APE1 + UDG. The hAAG concentration is 0.1 U/μL, and the UDG concentration is 0.1 U/μL. The 100 nM bifunctional dsDNA substrates and 2 U of APE1 were used in this research.

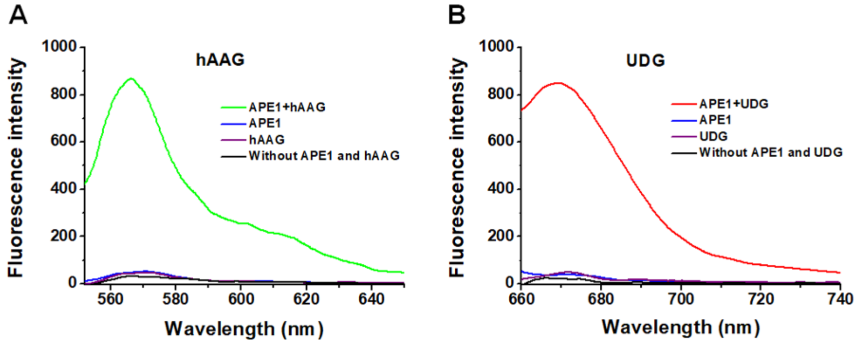

Fig. S5 (A) Measurement of fluorescence emission spectra of the hAAG-induced RCA products in different conditions including dsDNA substrate + APE1 + hAAG (green line), dsDNA substrate + APE1 (blue line), dsDNA substrate + hAAG (purple line), and dsDNA substrate (black line). (B) Measurement of fluorescence emission spectra of the UDG-induced RCA products in different conditions including dsDNA substrate + APE1 + UDG (red line), dsDNA substrate + APE1 (blue line), dsDNA substrate + UDG (purple line), and dsDNA substrate (black line).

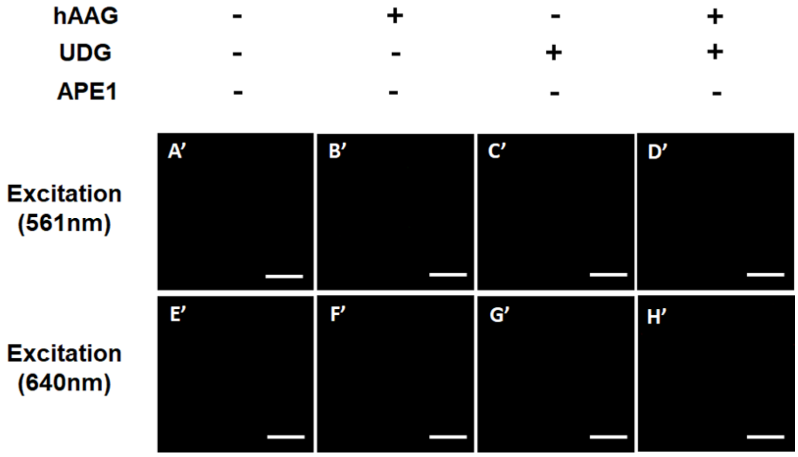

Fig. S6 Simultaneous detection of hAAG and UDG by TIRF-based single-molecule imaging in the absence of APE1. The Cy3 fluorescence signal is shown in green, and the Cy5 fluorescence signal is shown in red. The hAAG concentration is 1 × 10^{-3} U/μL, and the UDG concentration is 1 × 10^{-3} U/μL. The 100 nM bifunctional dsDNA substrates were used in this research. The scale bar is 5 μm. When APE1 does not exist, the Cy3 fluorescence signal

cannot be detected no matter whether hAAG exists or not (A′-D′), and the Cy5 fluorescence signal cannot be detected no matter whether UDG exists or not (E′-H′). These results demonstrate that APE1 is necessary for the cleavage of AP sites by hAAG and UDG (Fig. S1, step 2).

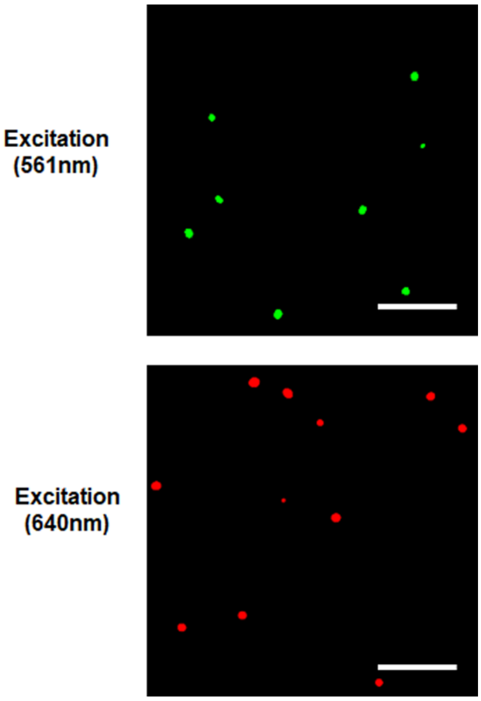

Fig. S7 Simultaneous detection of hAAG and UDG by TIRF-based single-molecule imaging. The Cy3 fluorescence signal is shown in green, and the Cy5 fluorescence signal is shown in red. The hAAG concentration is 1×10^{-11} U/μL, and the UDG concentration is 1×10^{-11} U/μL. The 100 nM bifunctional dsDNA substrates and 2 U of APE1 were used in this research. The scale bar is 5 μm.

Optimization of experimental conditions. To obtain the best assay performance, we optimized different experimental conditions including the concentrations of hAAG circular template and UDG circular template, the amount of phi29 DNA polymerase, the concentrations of Cy3-dCTP and Cy5-dGTP, the reaction time of RCA, and the concentrations of hAAG probe and UDG probe. We employed single-molecule detection to measure Cy3 and Cy5 counts for quantitative analysis. The concentrations of hAAG circular template and UDG circular template are the crucial factors which influence the amplification efficiency of RCA reaction. On one hand, the high-concentration circular template can lead to high RCA efficiency, but it might increase the RCA background signal correspondingly. On the other hand, the low-concentration circular template can decrease the background, but it may induce low amplification efficiency due to the lack of templates. We monitored the variance of Cy3 and

Cy5 counts in response to different concentrations of hAAG circular template and UDG circular template, respectively. As shown in Fig. S8, the counts of Cy3 and Cy5 improve with the increasing concentrations of hAAG circular template and UDG circular template from 1 nM to 50 nM, and reaches a plateau at the concentration of 50 nM, respectively. Thus, 50 nM is selected as the optimal concentrations of hAAG circular template and UDG circular template for RCA reaction, respectively.

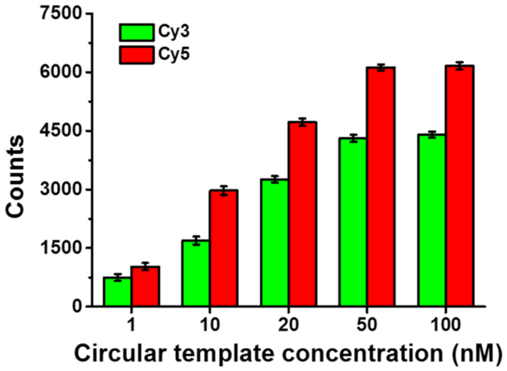

Fig. S8 Variance of the Cy3 counts in response to different concentrations of hAAG circular template in the presence of 0.1 U/μL hAAG (green column), and variance of the Cy5 counts in response to different concentrations of UDG circular template in the presence of 0.1 U/μL UDG (red column). Error bars show the standard deviation of three experiments.

In this assay, the RCA reaction relies on the amount of phi29 DNA polymerase, and thus the amount of phi29 DNA polymerase should be optimized. As shown in Fig. S9, the Cy3 and Cy5 counts enhance with the increasing amount of phi29 DNA polymerase from 1 to 5 U, and it tends to saturate beyond the amount of 5 U due to the exhaustion of hAAG primer, UDG primer, deoxyribonucleotides, or other raw materials. Therefore, 5 U of phi29 DNA polymerase is used in the subsequent researches.

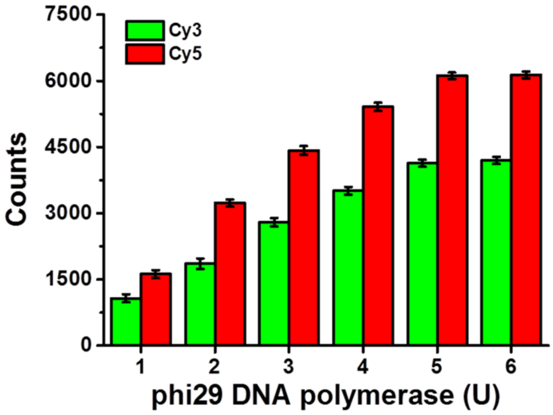

Fig. S9 Variance of Cy3 counts with the amount of phi29 DNA polymerase in the presence of 0.1 U/μL hAAG (green column), and variance of Cy5 counts with the amount of phi29 DNA polymerase in the presence of 0.1 U/μL UDG (red column). Error bars show the standard deviation of three experiments.

The Cy3-dCTP and Cy5-dGTP are the fuels of RCA reaction and may influence the amplification efficiency of RCA. To optimize the amounts of Cy3-dCTP and Cy5-dGTP, we monitored the variance of Cy3 and Cy5 counts in response to different concentrations of Cy3-dCTP and Cy5-dGTP, respectively. As shown in Fig. S10, the Cy3 and Cy5 counts enhance with the increasing concentrations of Cy3-dCTP and Cy5-dGTP from 2.5 to 10 μM, and reach a plateau at the concentration of 10 μM, respectively. This can be explained by the complete loss of phi29 DNA polymerase activity. Thus, we used 10 μM as the optimal Cy3-dCTP concentration and the optimal Cy5-dGTP concentration, respectively.

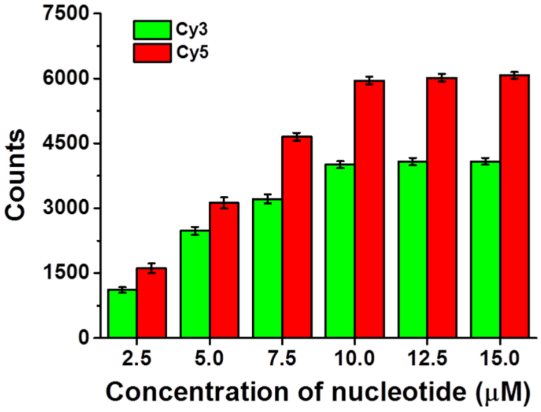

Fig. S10 Variance of Cy3 counts with the concentration of Cy3-dCTP in the presence of 0.1 U/μL hAAG (green column), and variance of Cy5 counts with the concentration of Cy5-dGTP in the presence of 0.1 U/μL UDG (red

column). Error bars show the standard deviation of three experiments.

We further investigated the effect of reaction time upon the assay performance. As shown in Fig. S11, Cy3 counts improve with the reaction time from 30 to 120 min, and reach a plateau at 120 min. The Cy5 counts enhance with the reaction time from 30 to 120 min, and it reaches a plateau at 120 min. This can be explained by either the complete loss of phi29 DNA polymerase activity or the consumption of all available deoxyribonucleotides. Thus, we used the reaction time of 120 min in the subsequent researches.

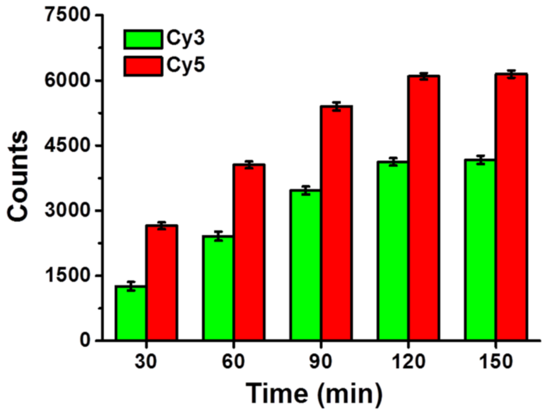

Fig. S11 Variance of Cy3 counts with the reaction time in the presence of 0.1 U/μL hAAG (green column), and variance of Cy5 counts with the reaction time in the presence of 0.1 U/μL UDG (red column). Error bars show the standard deviation of three experiments.

In the DNA glycosylases-induced excision reaction, hAAG probe and UDG probe are consumed. We further investigated the influence of the concentrations of hAAG probe and UDG probe upon the Cy3 counts and Cy5 counts, respectively. As shown in Fig. S12, the Cy3 counts enhance with the increasing concentration of hAAG probe, and reaches the maximum value at the concentration of 100 nM. Similar result is observed for the variance of Cy5 counts with the increasing concentration of UDG probe. Thus, 100 nM is selected as the optimal concentrations of hAAG probe and UDG probe, respectively.

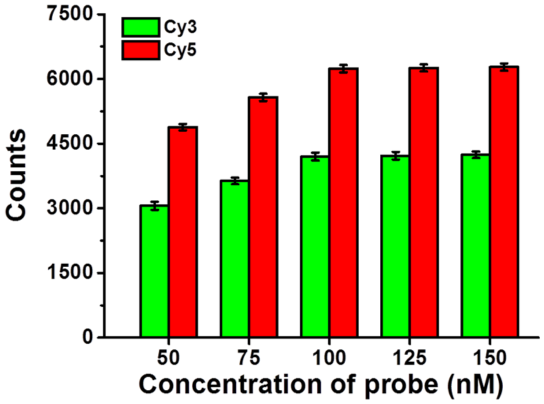

Fig. S12 Variance of Cy3 counts with the concentration of hAAG probe in the presence of 0.1 U/μL hAAG (green column), and variance of Cy5 counts with the concentration of UDG probe in the presence of 0.1 U/μL UDG (red column). Error bars show the standard deviation of three experiments.

References

1 P. J. O'Brien and T. Ellenberger, *Biochemistry*, 2003, **42**, 12418-12429.

2 D. M. Lyons and P. J. O'Brien, *J. Am. Chem. Soc.*, 2009, **131**, 17742-17743.
